# Should nasal respiratory support in preterm infants be tailored for physiologic maturity?

**DOI:** 10.1038/s41390-023-03016-z

**Published:** 2024-01-16

**Authors:** Manoj Biniwale, Rangasamy Ramanathan, Parviz Minoo

**Affiliations:** 1 Division of Neonatology, Department of Pediatrics, Keck School of Medicine, University of Southern California, Los Angeles, CA USA; 2 Hastings Center for Pulmonary Research, Los Angeles, CA 90033 USA

Newborn infants are born with various protective neurological reflex mechanisms. One of the main roles of larynx is to protect the airway from inhaling foreign matter. Laryngeal chemoreflexes (LCR) are airway protective reflexes that are triggered by stimulation of laryngeal mechanoreceptors in response to a number of stimuli including milk or gastric juices.^1^ Esophageal reflexes (ER) also known as aerodigestive protective reflexes contribute to protection of airways and lungs by forward propulsion of ingested food preventing reflux and aspiration.^2^ The most pronounced cardiorespiratory events occur when there is simultaneous stimulation of the proximal and distal esophagus.^3^ Both LCR and ER work together as the innervation of both pathways is shared through the vagus nerve to protect airways while ensuring safe feeding.

The study published by Elsedawi et al. in this issue of *Pediatric Research*^4^ compared the effects of LCR and ER in preterm lambs receiving various levels of nasal respiratory support (NRS) on the cardiorespiratory inhibition. LCR and ER were induced in the lambs under four experimental conditions: no respiratory support as control, nasal continuous positive airway pressure (nCPAP), high flow nasal cannula (HFNC) and HFNC with CPAP in a random order on day 6–7 and day 13–14 of life. Effects of these interventions on cardiorespiratory events such as apnea, bradycardia, cough, laryngospasm, and arousal from sleep were studied.^4^ Using the lamb as a model in a previous study, the authors showed consistently blunted LCR-related cardiorespiratory inhibition with nCPAP compared to caffeine which did not have any significant effects on LCR inhibition. In the present study the authors examined the use of nCPAP in LCR related apneas while validating the same model.^5^ Delivering lambs 14 days before term gestation corresponds to a similar physiologic maturity of 34 weeks of premature infants which is about the time when oral feedings are typically established. Also, studying the response at two time points a week apart is clinically relevant. As the preterm infants approach full term gestation the responses to these reflexes change due to physiologic maturity.

Even though NRS is known to decrease bronchopulmonary dysplasia (BPD), its effects on LCR an ER are not well understood. nCPAP provides a fixed amount of pressure to keep the upper airways open. HFNC provides high velocity gas with variable pressure delivery depending on the size of the cannula and ratio between nasal cannula size to nostrils. This promotes CO_2_ washout in naso-pharyngeal dead space and reduces inspiratory resistance in the upper airways. NRS using either nCPAP or HFNC is the preferred modality over invasive mechanical ventilation in preterm infants as it reduces the risks of problems, such as airway trauma or long term morbidities (e.g., BPD and neurodevelopmental disabilities).^6^ The work by Elsedawi et al. addresses these important points to determine whether increasing upper airway flow provided through HFNC or pressure provided by nCPAP attenuates LCR and ER responses, which typically contribute towards exacerbation of apnea and bradycardia and worsening of esophageal motility in preterm population. Furthermore, the present study tries to clarify whether the effects of nCPAP on upper airway and esophagus with higher set pressures may be different than HFNC with unpredictable lower or variable pressures.^4^

The findings of Elsedawi et al. study suggest a significant decrease in duration, percentage and number of episodes of bradycardia when lambs received nCPAP alone. Similar effects were not achieved by HFNC with or without nCPAP when compared to no respiratory support. On the contrary, HFNC led to more respiratory inhibition leading to desaturation and sustained laryngeal closure. It is interesting to note that these effects were significant only on day 6–7 and not on day 13–14. This probably is related to maturity of lambs over time blunting the reflex response to stimulation. Far more interesting is the persistence of inhibitory response after eliciting LCR in HFNC group with sustained laryngeal closure. This observation may be related to triple responses in SIDS hypothesis with a vulnerable infant, critical developmental period of homeostatic control and exogenous stressor such as high flow of air in these experiments.^7^ Clinically these findings have an important cautionary message for utilization of HFNC in preterm infants.

The study by Elsedawi et al. also found that in the first week of life LCR resulted in larger episodes of bradycardia, aspirations and apnea compared to ER. Surprisingly when elicited with ER, the lambs receiving nCPAP had increased respiratory inhibition both in the first and second week of life.^4^ While most of the previous studies with ER are focused on managing dysphagia with gastroesophageal reflux there have not been studies showing any relationship of ER with cardiopulmonary events in these settings with various levels of NRS support. Upper esophageal contraction reflexes and lower esophageal relaxation reflexes in preterm infants have been assessed in the past using nasal manometry catheter. Pressures were documented using mid-esophageal infusions of air as mechanosensitive stimulus and liquid as liquid sensitive stimulus. Effects of gestational age and postnatal maturation showed progression of inhibitory modulation with advancing maturation.^8^ The present study by Elsedawi et al. provides additional evidence to the findings of alteration of esophageal pressures secondary to flow of air through nCPAP, but, not with HFNC. Certainly, more studies are needed to assess the esophageal response to nCPAP to assess changes in ER.

Lastly, authors assessed significant differences in males compared to female lambs. Female fetuses typically have reduced airway resistance, more phospholipid production leading to more surfactant synthesis and have higher ratio of large to small airways. Gender differences in physiology, hormones, and growth factors may lead to females having better respiratory outcomes.^9^ Previous studies in rat pups showed LCR-induced apnea to be longer in stressed pups compared to controls with oxygen desaturation and bradycardia being more profound, especially in males. The latter was proposed to be related to disruption of neuroendocrine function by testosterone.^10^ These differences were not seen in male or female lambs having similar LCR and ER responses in the present study.

## Final thoughts

Clinical studies in LCR and ER are limited, especially when it comes to decision making for the mode of respiratory support. Decisions to provide the type of NRS in preterm infants after delivery is largely driven by the unit guidelines and individual experience of the providers rather than physiologic maturity of reflexes. Clinical trials are needed to assess the level of NRS required or changed using objective evidence of maturity of LCR and ER. While nCPAP may be helpful initially to blunt the response of LCR in very preterm infants, those infants showing exaggerated ER with gastroesophageal reflux may not benefit from nCPAP, with potentially worsening outcome. While HFNC may have a place in this subset of population, as it may not worsen the reflux, worsening of LCR with HFNC is very concerning.

Additional studies pertaining to the newer modalities of NRS support, such as nasal intermittent positive pressure ventilation and nasal high frequency ventilation are urgently needed. These modes not only provide sustained upper airway and esophageal pressures but add frequent pulsatile pressures to airway as well as esophagus. Whether that would exaggerate or blunt LCR remains to be determined. Hypercapnia activates the glottic dilator muscles and opens the larynx in newborn infants. Whether changes in carbon dioxide levels or nCPAP pressures would impact LCR or ER also remains a challenge that requires further investigation.
